# Malaria in Brazil: an overview

**DOI:** 10.1186/1475-2875-9-115

**Published:** 2010-04-30

**Authors:** Joseli Oliveira-Ferreira, Marcus VG Lacerda, Patrícia Brasil, José LB Ladislau, Pedro L Tauil, Cláudio Tadeu Daniel-Ribeiro

**Affiliations:** 1 Laboratório de Pesquisa em Malária, Instituto Oswaldo Cruz, Fiocruz, Pavilhão Leônidas Deane - 5° andar, Av. Brasil 4365, Manguinhos, Rio de Janeiro, RJ - CEP 21.045-900, RJ - Brazil; 2 Centro de Pesquisa Diagnóstico e Treinamento em Malária (CPD-Mal), Fiocruz and Secretaria de Vigilância em Saúde (SVS) - Ministério da Saúde (MS), Brazil; 3 Fundação de Medicina Tropical do Amazonas, Av. Pedro Teixeira 25, Manaus, Amazonas - CEP 69.040-000, Brazil; 4 Instituto de Pesquisa Clínica Evandro Chagas, Fiocruz, Av. Brasil 4365. Manguinhos, Rio de Janeiro, RJ - CEP 21.045-900, RJ - Brazil; 5 Programa Nacional de Controle da Malária, SVS-MS, Esplanada dos Ministérios, Bloco G, Sobreloja, sala 151. Brasília - CEP 70.058-900, Brazil; 6 Área de Medicina Social, Faculdade de Medicina, Universidade de Brasília, Brasília - CEP 70.910-900, Brazil; 7 Laboratório de Imunoparasitologia, Instituto Oswaldo Cruz, Fiocruz. Pavilhão Leônidas Deane - 4° andar. Av. Brasil 4365. Manguinhos, Rio de Janeiro, RJ - CEP 21.045-900, RJ - Brazil

## Abstract

Malaria is still a major public health problem in Brazil, with approximately 306 000 registered cases in 2009, but it is estimated that in the early 1940s, around six million cases of malaria occurred each year. As a result of the fight against the disease, the number of malaria cases decreased over the years and the smallest numbers of cases to-date were recorded in the 1960s. From the mid-1960s onwards, Brazil underwent a rapid and disorganized settlement process in the Amazon and this migratory movement led to a progressive increase in the number of reported cases. Although the main mosquito vector (*Anopheles darlingi*) is present in about 80% of the country, currently the incidence of malaria in Brazil is almost exclusively (99,8% of the cases) restricted to the region of the Amazon Basin, where a number of combined factors favors disease transmission and impair the use of standard control procedures. *Plasmodium vivax *accounts for 83,7% of registered cases, while *Plasmodium falciparum *is responsible for 16,3% and *Plasmodium malariae *is seldom observed. Although vivax malaria is thought to cause little mortality, compared to falciparum malaria, it accounts for much of the morbidity and for huge burdens on the prosperity of endemic communities. However, in the last few years a pattern of unusual clinical complications with fatal cases associated with *P. vivax *have been reported in Brazil and this is a matter of concern for Brazilian malariologists. In addition, the emergence of *P. vivax *strains resistant to chloroquine in some reports needs to be further investigated. In contrast, asymptomatic infection by *P. falciparum *and *P. vivax *has been detected in epidemiological studies in the states of Rondonia and Amazonas, indicating probably a pattern of clinical immunity in both autochthonous and migrant populations. Seropidemiological studies investigating the type of immune responses elicited in naturally-exposed populations to several malaria vaccine candidates in Brazilian populations have also been providing important information on whether immune responses specific to these antigens are generated in natural infections and their immunogenic potential as vaccine candidates. The present difficulties in reducing economic and social risk factors that determine the incidence of malaria in the Amazon Region render impracticable its elimination in the region. As a result, a malaria-integrated control effort - as a joint action on the part of the government and the population - directed towards the elimination or reduction of the risks of death or illness, is the direction adopted by the Brazilian government in the fight against the disease.

## Background

During the late 1930s, the Northeast Region of Brazil was invaded by *Anopheles gambiae *and a severe malaria outbreak, with a 13% fatality rate in a largely immune-naïve population, astonished Brazilian malariologists and health authorities. Because of the shipping traffic between Brazil and Senegal at that time, it was assumed that the invader came from this African region, probably in French warships travelling in 70 hours from Dakar to Natal to conduct meteorological studies preparatory of the transatlantic flights to be done in the future commercial companies [1]. Organized efforts to eliminate this very efficient malaria vector succeeded in the first years of the 1940s. The episode is considered to be one of the most important and successful Brazilian public health control campaigns.

**Figure 1:**
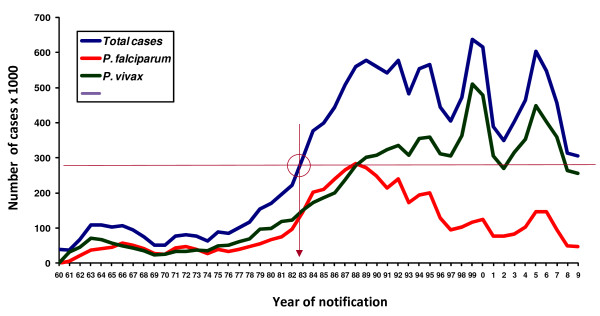
**Number of malaria cases registered yearly from 1960 to 2009 in the Brazilian Amazon according to the Plasmodium species responsible**. The great increase of malaria cases during late 70's and 80's was due to massive and uncontrolled migration to the Amazon region of people attracted by colonization programmes, sponsored by the government. The highest figures were recorded in 1999 (637 470 cases) when the government decided to implement the Plan for Intensification of Actions for Malaria Control (PIACM) in high-risk areas focusing on the early diagnosis and treatment of the cases. The circle with the intersection lines shows that the present number of malaria cases corresponds to the figures recorded in 1983. Notice also the progressively decreasing proportion of cases due to *P. falciparum *after 1988. In 1990 44.3% of cases were due to *P. falciparum *and the situation of the most prominent predominance of *P. vivax *transmission was reached in 1999 (only 18.6% of cases being due to *P. falciparum*).

It is estimated that in the early 1940s, malaria was a nationwide problem with around six million people, approximately 20% of the national population, infected each year [2]. However, during the late 1950s, a national and successful campaign, following the eradication aims dictated by WHO, gained strength in the country, decreasing malaria to its lowest level by 1960, when only 36,9 thousand cases were registered [3]. Although the eradication programme of the Ministry of Health in Brazil - based on DDT spraying on the walls inside the houses and the use of chloroquine to treat febrile cases - succeeded in freeing the majority of the country from malaria transmission by the late 1960s/beginning of the 1970s, it was, however, unable to contain the rapid spread of the disease in the Amazon Basin, where malaria still remains a serious health problem [4] (Figure 1).

From the mid-1960's onwards, Brazil underwent a rapid and disorganized settlement process in the Amazon, which witnessed a huge transformation. Colonization programmes, sponsored by the government, resulted in massive and uncontrolled migration and brought a new reality for which the area was not prepared. This migratory movement led to a progressive increase in the number of reported cases in the country that rose from 52 000 in 1970 to 578 000 in 1989 [3,5-7]. In 1992, the WHO sponsored a meeting on malaria control at Amsterdam (Netherlands), pushing Ministries of Health of countries where malaria was still endemic to adopt integrated malaria control measures focusing the decrease of malaria morbidity and fatality rather than its eradication [8].

The Brazilian National Programme for Malaria Control (Plano Nacional de Controle da Malária, PNCM), which had so far been based on DDT spraying in the intra-domicile of all houses of the endemic region (goal never achieved) and the diagnosis and treatment of present and recent febrile cases, reoriented its actions focusing them on early diagnosis and adequate treatment of cases. The policy of increasing the number of health posts able to perform these tasks started at that time and resulted, for the first time, in a decrease of the proportion of falciparum malaria cases (Figure 1). The highest figures were recorded in 1999 (637,470 cases), when the government decided to implement a plan to intensify the actions of the PIACM (Plano de Intensificação das Ações de Controle da Malária). The new plan had the main goals of reducing malaria incidence, morbidity (including the severe forms of the disease) and fatality; to eliminate malaria transmission in urban areas of the capitals of the Amazon States and to maintain the interruption of the disease transmission in places where this has been achieved. The Brazilian authorities also followed the recommendation of focusing the control strategies in the individuals rather than in the environment [9]. For this, the Secretary of Health Surveillance (SVS) of the Ministry of Health targeted an important expansion of the network of diagnosis/treatment stations in the Amazon. The figures were 1 182 diagnostic laboratories, 2,656 malaria control agents and around 2,4 × 10^6 ^blood examinations in 1999 as compared to the 3,492 diagnostic laboratories; 48,849 malaria control agents and around 2.8 × 10^6 ^blood examinations recorded in 2009 [10]. Apparently, the strategy of active case detection in some highly endemic areas, such as those in which Yanomami Indians were living, led to a sustained control of the disease [11]. However, the impact of this strategy by itself cannot be analyzed properly, because other interventions were applied at the same time as part of the PIACM, corroborating the idea that isolated interventions are not enough for the control [10].

**Figure 2:**
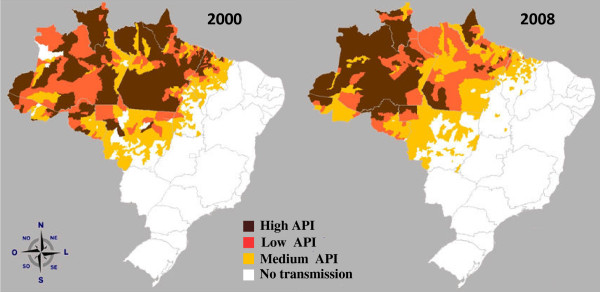
**Areas of malaria transmission in Brazil according to the Annual Parasitary Index (API) in the years of 2000 and 2008**. The Amazonian Annual Parasite Index (API, number of cases/thousand inhabitants) indicates success and failures of the control measures in some counties. Low API: < 10 cases; medium API: 10 to 49.9 cases; high API: > 50 cases. The distribution of transmission is not regular since 57 (7.1%) out of the 807 municipalities in the Amazon states concentrate 80% of the total registered malaria cases and only three (0.4%) of them accounts for 18% of the Amazonian cases.

However, a recrudescence of malaria transmission occurred in some localities of the Amazon and the incidence rose again from 2003 to 2005, bringing the figures to a situation almost comparable to that registered in 1999. The analysis of the reasons for this increase points to a multifactorial genesis of the phenomenon. This would involve climatic changes and migratory movements due to a disorderly occupation of the outskirts of large cities in the region as a result of agrarian reform projects and the consequent deforestation for logging, cattle ranching, agriculture, as well as for unofficial settlements. Poor performance in the implementation and administration of the actions prescribed by PNCM at the level of municipalities also contributed to increased transmission. Another contributor is the increase of the mosquito vector population as a result of an inadequate management of the environment. One example is the abandoned tanks used for fish-farming in the backyards of homes or on the outskirts of several towns in the Amazon region, as in Manaus, where they were common. To meet the new dynamics of transmission, the Ministry of Health initiated wide-ranging multi-sector mobilization of forces, mainly the health managers in states and municipalities in the Amazon region, to coordinate population movements and to prioritize surveillance, prevention and control of malaria on their agendas. The effects of this joint effort are reflected in a substantial reduction of cases from 2006 onwards. The number of cases fell thereafter to 456 000 in 2007. The last available data show that, in 2008, an additional 31% reduction in the number of cases brought the figures (314,420 cases) to a situation comparable to that of 1983 (Figure 1). The Amazonian Annual Parasitological Index (API, number of cases/thousand inhabitants) fell also from 31.9 in 1999 to 12.8 in 2008 [10]. In order to take advantage of this kind of results and information to improve and consolidate the malaria control actions, it is necessary to try to analyze and understand results that indicate success, as is done when facing failures of the control measures and worsening of the malaria situation. The Coordination of the National Control Programme considers that the strengthening of the local management capabilities, through the continued expansion of the diagnosis and treatment network, may account for the sustained reduction of cases from 2006 onwards. This produced a decrease in the rates of disease severity as well as the reduction of the numbers of municipalities at (high, medium and low) risk in the Amazon (Figure 2). According to the PNCM, the absence of such approaches may explain the concentration of cases in some counties, where management has not prioritized the control of malaria, or other conditioning factors.

It is also important to point out that, although *Anopheles darlingi*, the main mosquito vector, is present in about 80% of the country, the incidence of malaria in Brazil is almost exclusively (99.8% of the total number of cases) restricted to the Amazon Region, where a number of combined factors favor disease transmission and impair the use of standard control procedures (Figure 2)[3]. The number of cases in the nine States of the Brazilian Amazon has fluctuated over the years, increasing in some states where new settlements projects and gold mining activities are implemented. In addition, the transmission inside the region is not regularly distributed, since 57 (7.1%) out of the 807 municipalities in the Amazon states account for 80% of the total registered malaria cases and three of these accounts for 18% of the Amazonian cases [10]. In addition, and unfortunately, the disease seems to be concentrated not only in localities, but also in some groups of individuals. Although the overall incidence of malaria is decreasing, an augmented proportion of the disease in women (34,9% in 2003 to 38.6% in 2008) and children below 10 years of age (22,0% in 2003 to 25,2% in 2008) has been recorded in the last years [10]. The reasons remain to be determined but environmental factors, such as proximity of houses to mosquito-breeding places, could be involved. A follow-up of a cohort of around 6,400 women at the Coari Municipality (Amazonas - Brazil) in 2001 and 2002 has shown that only 0.1% of parasitologically-negative women and 92% of the positive ones reported at least one past episode of malaria[12]. The economical development of the new borders in the Northern Brazil had a great impact and could explain the maintenance of the disease in cities like Manaus, a city of two-million inhabitants, with a tax-free status, which attracts thousands of workers from several parts of the country and abroad [13].

Any decision-making process depends on obtaining robust data on the parameters to be monitored. For this, it is necessary to implement an epidemiological surveillance system and to permanently strengthen and improve local structures to generate and record standardized information that can guide the decisions, both at a local and national level. One positive aspect of the PNCM is the way the management of epidemiological information and the consolidation of routine work are conducted. Its information system (SIVEP-Malaria) is now highly developed and operates with a high coverage. All cases of malaria are included individually in a national database, with inputs from a network at the local level. The information recorded in the system is sent rapidly, via the web, to the regional levels and to the headquarters of the PNCM in Brasília. Automated reports are available on-line permanently, with input of approximately 42% of cases in eight days and 82% of cases in less than 30 days after the end of the corresponding month. Reports produced after 60 days of the period closure may cover more than 95% of cases. The SIVEP-Malaria database contains variables that allow the evaluation and selection of operational and epidemiological indicators, for which there are already pre-established routine analysis by the PNCM, and that are useful for monitoring and evaluating the situation. The system also allows monitoring of the scattering parameters of transmission in each city, which is useful to evaluate the degree of coverage of the diagnosis, treatment and use of insecticide-treated bed nets. Furthermore, the diagnosis offer versus the demand of cases can also be analyzed, in order to guide and adjust the diagnosis network. SIVEP provides also data related to the dispensing of drugs and the proportion of treatment failures among the diagnosed cases. The simplicity of the form guarantees a good quality of data and it is estimated that the system has a good sensitivity, since all the medication to treat malaria is distributed free by the Ministry of Health, through the reporting of cases. Nevertheless, the localized analysis of the system peculiarities in each municipality makes it possible to identify a heterogeneity of the general characteristics.

A project, recently submitted by the Brazilian Government (PNCM-Ministry of Health) and approved by the Global Fund, focuses the investment in epidemiological intelligence in strategic localities in the Brazilian Amazon and has the potential to change the history of malaria in Brazil. The goal of the project is to support and improve the capacity of local health services in order to enhance the understanding of the dynamics of the disease transmission and to conduct, with greater efficiency, the management of the intervention measures of the project and the control actions of the regular programme. The project aims to decrease morbidity and mortality in the Amazon region and the main expected result in the five-year project is a 50% reduction in the number of malaria cases in the 47 municipalities of the Amazon region, which had accounted for 70% of malaria cases in Brazil in 2007. The methodology is based on the strengthening of two recognized intervention measures: a) ensure early diagnosis and the administration of a timely treatment with effective drugs, amplifying the diagnosis network and improving the drug management, and b) quickly achieve high coverage of prevention with long-term insecticide-treated bed nets. The project is committed to concentrate in epidemiological intelligence and efficient management at the local level. This would be based on the establishment of a routine analysis for decision-making in close coordination with operating and management systems at all government levels (states and municipalities). This would also promote community organization and mobilization to facilitate participation in specific malaria control actions and improve acceptance of control measures by the communities.

In addition, we must emphasize the Brazilian capabilities of large-scale manufacturing of antimalarials. For instance, the association of artesunate/mefloquine produced by Farmanguinhos (Fiocruz) after a joint action of the Brazilian Ministry of Health and the Drugs for Neglected Diseases iniative (DNDi), is presented in dosages for adults and children, therefore facilitating drug intake and increasing adhesion to treatment. Currently, the State of Acre, in the Amazon, and all the States of the Extra-Amazonian Region successfully use the drug.

It is important to point out that the use of tools, such as artemisinin-based combination therapy, impregnated bed nets and rapid diagnostic tests, have not yet been evaluated in an integrated manner in a horizontal health system, such as the one offered in the Brazilian Amazon. This may indicate that the elimination of the disease in the country can be effectively achieved in the future. On the other hand, the analysis of available data, when the figures of malaria in Brazil achieve the situation of residual transmission in the future, may indicate the need of readapting the PNCM policy in view of the elimination of the disease in the Amazon Region and in the whole country.

Although the PNCM has succeeded in reducing drastically deaths, severe cases, hospitalizations and the national incidence of the disease, it seems important to consider its vulnerability, as the programme is based fundamentally on the diagnosis and treatment. In view of the potential emergence of *P. falciparum *resistance to artemisinin-based combination therapy and the absence of alternatives drugs, research programmes to identify new drugs that may replace the present ones are extremely important and urgently needed.

### The changing pattern of *P. falciparum and P. vivax *transmission

In Brazil, malaria is caused by three species of Plasmodium: *P. vivax *(that accounts for 83,7% of the registered cases), *P. falciparum *(causing 16,3% of the cases) and *P. malariae *(a small proportion of cases). No autochthonous transmission of *P. ovale *and *P. knowlesi *occurs [10]. Leônidas de Mello Deane, one of the most important Brazilian and world malariologists, used to say that the Superintendência de Campanhas (SUCAM, the national organization in charge of malaria control in Brazil, until its fusion with the *Fundação Serviços Especiais de Saúde Pública*, SESP, giving place to the *Fundação Nacional de Saúde *- FNS or FUNASA) had "eradicated" *P. malariae *from Brazil in the 1980s simply by switching the method of diagnosis from thin to thick smear, the only official method for malaria diagnosis in Brazil. Indeed, with this procedure it is not possible to assess the morphology of infected red blood cells and the parasite-altered shape can lead to a mistaken identification of *P. malariae *as *P. vivax*. Therefore, the failure to report *P. malariae *in endemic areas is not surprising. Studies on antibody response to *P. malariae *CS protein and the use of nested polymerase chain reaction suggest that this parasite could possibly be more prevalent in scattered areas, at least in the populations studied in Rondônia, Amazonas and Pará States [14,15]. However, no case of *P. malariae *infection was found by PCR among febrile patients in Manaus [16].

**Figure 3:**
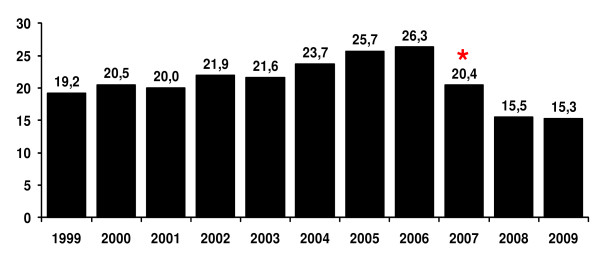
**Evolution of the proportion of malaria cases due to *Plasmodium falciparum *in the Amazon from the year of 1999 to 2009**. Since *P. falciparum *gametocytes only appear in the peripheral blood after 8 to 10 days of infection, prompt diagnosis and adequate treatment of the *P. falciparum *malaria cases can interrupt parasite transmission more efficiently than it can do in the case of *P. vivax *malaria, in which gametocytes are already present in the circulation in the first three days of blood infection [15]. * Beginning of the new therapy artemether/lumefantrine, the first line regimen for non-complicated falciparum malaria in Brazil. Source: Sivep - Malaria - Data updated on September 9th, 2008.

Even if considering that the incidence of *P. malariae *in Brazil may be underestimated, it is worth mentioning that predominant incidence of *P. vivax *malaria is a quite recent phenomenon (since the early 1990s) and results from, or has been at least very probably reinforced by, the pressure exerted by the PNCM aiming at the early diagnosis and treatment of cases. Indeed, since *P. falciparum *gametocytes appear in the peripheral blood only after 8 to 10 days of infection, prompt diagnosis and adequate treatment of the falciparum malaria cases can interrupt parasite transmission more efficiently, than in the case of *P. vivax *malaria, in which gametocytes are already present in the circulation in the first three days of blood infection [17]. Thus, the relative incidence of the two main *Plasmodium *species transmitted in Brazil was around 50% each in 1988. This changed from 1990 onwards (when 44.3% of cases were due to *P. falciparum*), reaching the situation in 2009 when *P. vivax *has become the predominant species (only 16,3% of cases being due to *P. falciparum*)(Figure 3).

### The decreasing occurrence of severe malaria in Brazil

**Figure 4:**
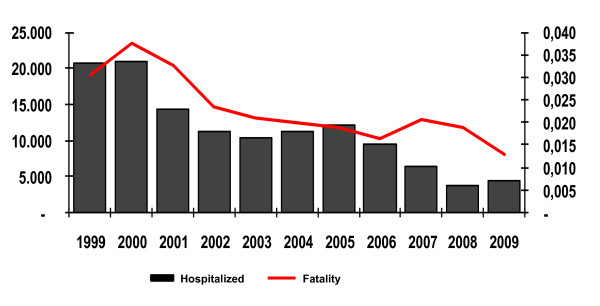
**Malaria fatality rates and number of hospitalizations due to the disease in the Brazilian Amazon from 1999 to 2009**. The number of hospitalizations due to malaria in the Brazilian Amazon dropped from 53,450 in 1994 to 18,037 in 2000 and 4,442 in 2009 as did the number of registered deaths attributed to the disease (from 897 in 1984 to 58 in 2009) as well as the fatality rate (from 0.038% in 2000 to 0.013% in 2009). The malaria cases seen in Brazil are being rapidly diagnosed and treated (59% of all malaria cases in 2008 registered in the Amazon were treated in the first 48 hours after appearance of symptoms, Malaria/SVS/MS, 2009), diminishing both the transmission and the occurrence of severe cases.

*Plasmodium vivax *is thought to cause little mortality but, as *P. falciparum*, it accounts for a vast amount of morbidity and for a huge burden on the prosperity of endemic communities. Probably as a result of the early diagnosis and treatment of the cases and of the decrease in *P. falciparum *transmission, the number of hospitalizations due to malaria dropped (53,450 in 1994 to 18 037 in 2000 and 4,442 in 2009) as did the number of registered deaths attributed to the disease (from 897 in 1984 to 58 in 2009), together with the fatality rate (from 0.038% in 2000 to 0.013% in 2009) in the Amazon (Figure 4). This is probably a direct result of the early diagnosis and treatment of the malaria cases as a consequence of the already mentioned expansion of the network of Laboratory and Health Agents in the Amazon. In fact, 59% of all malaria cases registered in 2008 in the region were treated in the first 48 hours after appearance of symptoms [10], diminishing both the transmission and the occurrence of complicated cases in endemic areas.

In one illustrative example of the present situation, as far as malaria morbidity in Brazil is concerned, Fernandes *et al *[18] studied 127 *P. falciparum *and 74 *P. vivax *malaria patients in two localities (Belém and Paragominas) of the Amazonian State of Pará and could find only one case of severe anaemia (associated to *P. vivax *and not to *P. falciparum *infection). No other malaria patient enrolled in the study was hospitalized due to anaemia or for any other reason. The low frequency of hospitalization observed during the period of the study (2001 to 2003) was in accordance with the overall picture recorded for the state of Pará in the same period, when there was a sharp reduction in the total number of malaria cases (279,303 in 2000 and 123,490 in 2003) and in the frequency of hospitalization (3.3% in 2000 and 2.6% in 2003) [19]. The absolute number of hospitalized patients from 2000 to 2003 in Belém dropped drastically as well (140 hospitalized patients in 2000 and 49 in 2003) and particularly in Paragominas (275 in 2000 and only six in 2003). In the same way, the mortality due to malaria in 2001 to 2003 was low, with a total of 119 fatal cases out of 458,305 malaria cases [0.26 fatal outcomes per 1 000 malaria cases, or 10.2 deaths per 1 000 hospitalized patients [Rui Bráz and Carlos José Mangabeira-Silva (PNCM-SVS/MS), personal communication]. Similar data were presented by Caicedo *et al *[20], who found that severe anaemia was rare amongst patients from two distinct malaria endemic areas in Manaus (Brazil) and Tumaco (Colombia).

In spite of the decreasing severity of malaria observed in Brazil in the last years, one point that merits to be emphasized, is that, precisely as a result of the concentration of malaria in the Amazon region, general practitioners outside the Amazon region are much less alert to consider the diagnosis of malaria, when facing a febrile patient. Migration and international travel require personnel with specific expertise, highlighting the importance of the specialty of Infectious Diseases and the sentinel surveillance of febrile travelers. One illustrative example would be the already reported frequent confusion of malaria with dengue in the city of Rio de Janeiro [21]. Visitors from Africa might die of falciparum malaria about seven days after the beginning of the symptomatology, with more than half of all erythrocytes parasitized [22]. These are probably the reasons why malaria lethality is more than seventy times higher in this region (1,27%) than in the Amazon (0,018%)[10]. It is noteworthy that most of the travelers' clinics in Brazil are concentrated in Rio de Janeiro and São Paulo states.

In summary, the inversion of the *P. falciparum/P. vivax *cases ratio in Brazil in the last two decades was a major achievement of the National Control Programme, leading to a substantial decrease in the number of deaths. However, this may be troublesome regarding the future perspectives of eliminating malaria in Brazil, since policy-makers are less prone to privilege investments in a disease with low fatality rates and with a massive incidence outside the economic axis area of the country. Some neglected effects of the 'benign' *P. vivax *infection must be considered because they may compromise the development of endemic countries, such as the impact upon school performance, as recently shown in children from an endemic area in the Amazonas State [23]. In addition, vivax malaria may not always be benign [10].

### Unusual clinical complications associated with *P. vivax *in the Brazilian Amazon

*Plasmodium vivax *infection is generally recognized in the literature as a benign disease, despite triggering fever with a lower peripheral parasitaemia, as compared to *P. falciparum *[24]. However, in the last few years a pattern of unusual clinical complications with fatal cases associated with *P. vivax *have been reported in Brazil and is a matter of concern for Brazilian malariologists [25,26]. It is difficult to assume that there has been a worldwide increase of severity associated with *P. vivax *infection. Although most of the publications had previously been biased towards *P. falciparum *infection and its associated severity in the African continent, where most of the deaths are concentrated, more recently some attention has been devoted to the burden of the complicated *P. vivax *disease in Southeast Asia [27] and Latin America [28].

In Brazil, importantly and curiously, paralleling the increase in the proportion of *P. vivax *malaria recorded from the middle of 1980s to the beginning of the 1990s, an increase in the frequency of unusual clinical complications in *P. vivax *infected patients has been observed in the Amazon. According to the official statistics, from 1998 to 2008, 234 deaths related to *P. vivax *infection were reported in the Brazilian Amazon [10,29]. However, one must keep in mind that the description of a severe case and, even more, of a fatal case of *P. vivax *malaria requires, as a *sine qua non *condition, the confirmation of the involved species using a highly sensitive approach (*e.g*., PCR) to eliminate the possibility of a mixed (*P. falciparum/P. vivax*) infection and to rule out the presence of other simultaneous acute infectious diseases (*e.g*., dengue, yellow fever and other Amazonian virus - such as Oropoupouch and Mayaro, leptospirosis, typhoid fever, sepsis), as well as other chronic diseases, such as sickle cell anaemia, which may decompensate as a result of the *P. vivax *infection [25]. Likewise, fatal cases must ideally be submitted to a full autopsy allowing the characterization of its major anatomo-pathological findings. Despite representing a very low fatality rate, such severe cases were not reported when *P. falciparum *was the predominant species in Brazil. They may be associated to an increased exposure of non-immune population to this species, leading to the occurrence of severe *P. vivax *infection, similar to what was observed in the north-west of India, in Rajhastan, where severe *P. vivax *cases started to show up only after the inversion in the *P. falciparum/P. vivax *cases ratio [30,31].

The proportion of hospitalized cases due to *P. falciparum *decreased from 29% in 2003 to 25% in 2008, those due to an increase of *P. vivax *from 38% to 49% [10]. While the number of lethal cases of vivax malaria is stable since 2001 (around 21 cases/annum), the decrease in malaria transmission and in the absolute number of registered cases of vivax malaria since 2005 result in a mild but regular increase in the fatality rate in the last years, the highest rate having been observed in 2008 (0.008%)(Figure 4).

Manaus, the capital of the Amazonas State, is one of the three municipalities that, together with Porto Velho (Rondônia State) and Cruzeiro do Sul (Acre State) accounted for about one fifth of the total number of cases notified in Brazil in 2008 [10]. In Manaus, the Fundação de Medicina Tropical do Amazonas (FMT-AM) - that serves as a reference centre for health care and research on tropical diseases and diagnoses - treats around 30% of the malaria cases seen in this municipality. In Manaus, the total number of hospitalized *P. vivax *malaria cases as well as the proportion of the hospitalized cases due to this species (in relation to *P. falciparum*) seems to be increasing in the FMT-AM [32]. The major complication found in patients with *P. vivax *infection is thrombocytopenia, which accounts for 20% of the admissions to this tertiary care hospital in Manaus [26,33]. In the majority of the cases, severe thrombocytopenia (< 50 000 platelets/mm^3^) is not associated with coagulation disorders, such as disseminated intravascular coagulation [34]. In Manaus, severe thrombocytopenia was found in 8.9% of the patients with *P. vivax *infection, with only bleeding in one fourth of them (Lacerda MVG, unpublished data). The clinical relevance of this haematological complication, however, needs further clinical studies, as well as for *P. falciparum*, since there is no reported fatal case of malaria presenting exclusively with severe thrombocytopenia. Other relevant clinical complications include patients developing immune thrombocytopenic purpura after curing the infection [35] and splenic haematoma [36]. In non-endemic areas of malaria in Brazil, such as São Paulo, severe cases of *P. vivax *infection are being reported in travelers [37], and thrombocytopenia is often misattributed to dengue infection in Rio de Janeiro, where the disease is endemic [38].

At the FMT-AM, in a retrospective study from 2001 to 2002, applying the WHO criteria of malaria severity (traditionally used for *P. falciparum *malaria), 12.8% of the patients were diagnosed as having severe *P. vivax *malaria (43 out of 336 patients hospitalized with *P. vivax *malaria during the period). The clinical complications were very similar to those associated with *P. falciparum *infection, *e.g.*, hyperbilirrubinaemia, severe anaemia, acute renal failure, pulmonary edema and algid malaria [39]. In a series of cases with the parasitological diagnosis of *P. vivax *infection, which evolved to death in the same institution and were submitted to a full autopsy, the major findings were acute tubular necrosis, pulmonary edema and pneumonia (Lacerda MVG, unpublished data). Most of the patients who died presented other co-morbidity that could have contributed to the death, such as chronic liver disease, cardiac disease and G6PD deficiency. Series of severe cases have also been reported in other endemic areas for *P. vivax *malaria [30,40], but no standardized criteria are being routinely used for the clinical description of such cases in the literature.

Although this is not in the scope of this overview, it is important to briefly consider some aspects of the treatment of *P. vivax *malaria, which is no longer a simple task in many parts of the world, including Brazil. For instance, the radical cure of hypnozoites with primaquine (the only hypnozoiticidal drug currently approved for clinical use), almost always feasible with the shorter regimen of 30 mg/day for seven days in adults currently used un Brazil, may require the classical long-term (14 day) regimen [41]. With the increase in the number of *P. vivax *infections, the widespread use of primaquine for the radical cure may trigger severe complications (such as haemolysis and methaemoglobinaemia) in patients deficient for G6PD, which frequency is around 3% among males in Manaus [42]. These complications can be mistaken as a complication of *P. vivax per se *[43].

There is also *in vivo *evidence for the resistance of *P. vivax *to chloroquine in the Brazilian Amazon [44]. The resistance to chloroquine was firstly reported in Manaus in 1999 [45] and more recent data, from studies conducted in the context of the RAVREDA (Amazonian Network for Surveillance for Resistance to Antimalarial Drugs) with a proper follow-up of patients exclusively using chloroquine and in whom the drug plasma concentration was performed, seem to confirm such an observation [46]. However, since chloroquine plus primaquine, the drug association used for the radical cure of *P. vivax *infection, have a synergistic action [47], further studies are needed before any definitive conclusion can be proposed. Such studies should include non-endemic areas where the chance of reinfection is minimal and the potential for monitoring real resistance to both drugs is enhanced.

### Malaria outside the Amazon region and the "bromeliad-malaria"

**Figure 5:**
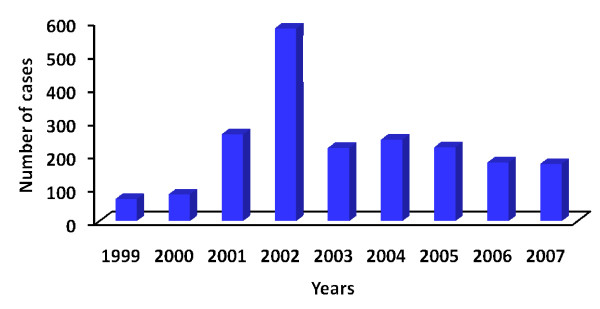
**Number of cases of autochthonous malaria seen outside the Amazon region**. The peak registered in 2002 is due to an outbreak of *Plasmodium falciparum *malaria observed in the State of Ceará, Northwestern Brazil. Cases seen outside the Amazon are mainly due to the transmission by *Anopheles (Kertezia) *occurring in the Atlantic forest possibly maintained by the involvement of infection of monkeys.

As an expected consequence of the existence of some thousands of malaria cases in the Amazon basin, a few cases do occur also outside the area of active transmission. Some of them correspond to autochthonous cases of malaria transmitted and maintained in small foci of transmission with very specific characteristics, such as those reported in the Atlantic Forest. The historical series of autochthonous cases outside the Amazon region is presented in Figure 5, showing a huge increase in the numbers in 2002 due to an outbreak of *P. falciparum *malaria occurred in the Ceará State, north-eastern Brazil.

Considering the State of Rio de Janeiro, where the headquarters of Fundação Oswaldo Cruz and CPD-Mal (the Reference Center for Malaria at the Secretary of Health Surveillance at the Ministry of Health) are located, a total of 1,505 malaria cases were reported from 1990 to 2008 (79 cases/year). Rio de Janeiro is the most visited city in Brazil (two million of tourists annually) and the home of 20% of all Brazilians who travel abroad for leisure or business. Probably as a result of this, most of malaria cases seen in the city are expected to be imported from the Amazon region and Africa. However, in the 2001-2008 period, 30% of the 29 reported autochthonous cases in Rio de Janeiro originated from Nova Friburgo, a mountain region in the Atlantic Forest. In the 2006-2008 period, three cases, diagnosed (by thick blood Smear, and PCR) as *P. vivax *malaria, came from localities situated between the municipalities of Nova Friburgo and Guapimirim at the "Serra dos Órgãos", also in the Atlantic Region. Patients presented at the Acute Febrile Diseases Outpatient Clinics of the Instituto de Pesquisa Clinica Evandro Chagas (a branch of the CPD-Mal at Fiocruz) with low parasite (*P. vivax*) counts and mild symptoms including low-grade (< 38°C) fever and splenomegaly [38]. Mild atypical symptomatology and very low parasitaemia was also the predominant presenting form of the disease in the autochthonous *P. vivax *malaria reported in inhabitants of two parks in the Atlantic forest of the São Paulo State between 1990 and 2000 [48,49]. Little is known about the factors involved in the chain of native transmission of malaria in the Atlantic region and malaria outbreaks in the Atlantic forest of Rio de Janeiro are not new events. Serological and entomological surveys performed in 1993 and 1996 in a village situated in a mountain valley: Rio Bonito in Rio de Janeiro state, where an outbreak of vivax malaria had occurred, suggested that *Anopheles (Kerteszia) cruzii *was a potential vector of malaria in this region [50]. At that time, one of the hypotheses was that members of a group of religious sects originating from the Amazon and constantly visiting the Amazon region could be responsible for the introduction of malaria in the district of Lumiar (Rio de Janeiro). The installation of a timber factory in Friburgo, working with products of trees brought from the Amazon region, points to another possibility for carrying mosquitoes as foci of infection.

**Figure 6:**
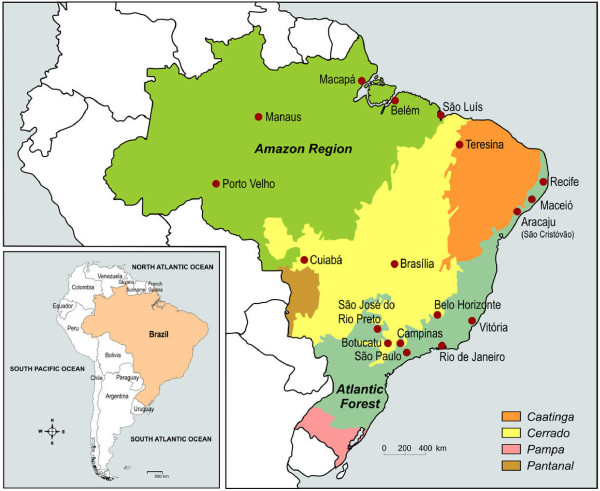
**The Brazilian Biome Map and location of Research Centres**. The Amazon region covers 40% of the surface of South America and 5% of the surface of the world, and 61% of its territory is in Brazil. It has the largest hydrographic network on earth, covering 20% of the worlds fresh water reserves and shelter the largest number of life forms on earth; the Atlantic forest (seasonal semideciduous forests) is the third largest Brazilian biome that stretches for about 4 000 km along the Atlantic coast between Rio Grande do Norte and Rio Grande do Sul; the "Caatinga" (semiarid steppe of Northeast Brazil) is located on the north-east coast is the largest dry forest region in South America and characterized by a semi-arid climate, low and unregular rainfall, fertile soils and an apparently dry vegetation; the Cerrado (Brazilian Savanna) is located on the large plateau that occupies the central highlands. Influenced by the four surrounding biomes, the fauna and flora of the Cerrado is extremely rich and the climate is hot and semi-humid; the Pantanal (Brazilian wetlands) is located in the watershed of the Upper Paraguay Rivers the largest area of fresh water marshes in the world, shared between Brazil (60%), Bolivia and Paraguay. The Pampas are the fertile South American lowlands in the southernmost end of Brazil, the climate is mild and contain unique wildlife because of the different terrains around it.

Deane [51] suggested that simian *Plasmodium *species could be responsible for these cases of malaria known as "bromeliad-malaria" in forest areas, where monkeys of the genera Allouata and Cebus and the *Anopheles (Kertesia) cruzii *and *Anopheles (Kertezia) bellator*, which breed in the water collections formed inside the bromeliads, are present. The presence of malaria in people coming into such areas of dense forest could ensure both simian and human malaria transmission. Duarte and coworkers [52] showed the occurrence of malaria parasites and serological responses against asexual forms and synthetic peptides mimicking the immunodominant epitope of the circumsporozoite protein (CS protein) of human malaria parasites in wild monkeys from areas of autochthonous human malaria. The existence of *P. vivax *variants and simian malaria in the Atlantic forest of Rio de Janeiro and Espirito Santo States also points to the possibility that monkeys could be the natural reservoir for malaria in the Atlantic forest and explain the autochthonous cases registered (Figure 6)[48,50,53]. Recent studies have shown that the simian malaria *P. knowlesi *in the human host is not rare, is widely distributed and can result in severe disease and death in humans, particularly in areas inhabited by the natural macaque host [54]. Taken together these data support the view that malaria transmission from neotropical primates to humans, and vice versa, may also occur more frequently than currently believed, both in the Amazonian rainforest and in the Atlantic forest.

The interruption of transmission of the "bromeliad-malaria", which occurred during the years 1980 to 1982 in the state of Santa Catarina - southern Brazil, was important and a good example that alternative control measures may be required to the control of residual malaria in an given area [55]. The transmission was restricted to two municipalities (São Francisco do Sul and Taquari) in that state and had never been interrupted, despite many efforts. In these two municipalities with a population of thirty thousand people, one thousand and six hundred autochthonous cases occurred each year, mainly in adults males under 50 years old. The transmission was seasonal, from November to April and all cases were due to *P. vivax*. The main vector was *An. cruzii*, which usually bites animals and humans outside houses. The use of DDT spraying inside the houses - a traditional measure in the control of malaria - was rejected by a high proportion of people, because its ineffectiveness, and because other interventions previously used, such as mechanical and chemical massive destruction of bromeliads, were also ineffective. The hypothesis that monkeys could be an eventual reservoir maintaining the parasites from May to October was discarded since they were not found in the area. Therefore, other hypotheses were considered to explain the maintenance of the circulation of parasite in humans in those localities. They included the presence of either asymptomatic *P. vivax *infections - patients with very low parasitaemia undiagnosed by microscopic examination - or of a *P. vivax *strain (already described in temperate climates) with long-term incubation period. A sero-epidemiological survey was performed with the aim of identifying parasite-carriers needing to be treated [56]. In 1980, around twenty seven thousand blood samples were collected on filter paper and sent to be processed in the SUCEN Laboratory at São Paulo. About five hundred people presented antibody (as revealed by anti-human total Ig antibodies) titres over 1:64 and were treated with chloroquine plus primaquine. In the following year, only twenty cases were diagnosed. In 1982, the sero-epidemiological survey was repeated, and two hundred cases were considered positive, with IgM/IgG over 1:64. They were treated and until now, about 27 years later, no more autochthonous cases were registered in the area.

If, on the one hand, studies conducted in the neighbouring states of São Paulo and Espírito Santo confirmed, using serological or molecular approaches, the asymptomatic infection by *Plasmodium *in areas outside the Amazon region, the lack of investigation of indigenous malaria cases in the state of Rio de Janeiro, on the other hand, does not give any information on what actually happens in the region (Figure 6). Further investigations are needed to confirm the presence of asymptomatic *Plasmodium*-infected individuals in the Atlantic region, since transfusion malaria is rarely diagnosed, but does occur and can become an important concern to medical services and health authorities [57].

Finally, it is important to emphasize to those involved in giving international travel advice that, excepting for the examples given above, which concern specific localities of the Atlantic forest and must be considered rare events, malaria transmission does not occur outside the Brazilian Amazon. Therefore, anti-malarial chemoprophylaxis is neither needed nor recommended by the Brazilian Ministry of Health or the WHO to those visiting exclusively areas outside the Amazon Region. Thus, the visitors attending the Olympic Games of Rio de Janeiro in 2016 will not need to be submitted to the side effects of anti-malarials throughout their stay in the city.

### Some information arisen from the study of the naturally acquired immunity

The pattern of malaria transmission in Latin America is different from that in the endemic areas of Africa. In Brazil, malaria is endemic in the Amazon region and is frequently associated with migration movements of non-immune individuals to areas where malaria is endemic. The population exposed to malaria in these areas, regardless of age, is vulnerable and infections tend to be followed by clinical symptoms [6,58]. For a long time, asymptomatic cases have been considered rare in Brazil [59]. However, asymptomatic infection by *P. falciparum *and *P. vivax *were detected in studies in the states of Rondonia and Amazonas, suggesting that subjects exposed to malaria in Brazil also develop acquired resistance to clinical malaria despite the epidemiological profile different from the one seen in Africa [60-63].

It is well-established that acquired clinical immunity to *P. falciparum *malaria depends on long-term repeated exposure to the parasite, a conclusion based on the observation that effective natural immunity to this species is restricted to areas of high level of transmission and endemicity, where age has always been considered to reflect the degree of exposure. It is now known, however, that non-immune adult migrants can naturally acquire reasonable clinical immunity more rapidly and efficiently than children, probably reflecting a different maturity of the immune system [64].

Studies on acquired immune responses against *P. falciparum *of individuals naturally-exposed who live in endemic areas of Brazil show that individuals primed against *P. falciparum *in their natural habitat, present a very diverse array of responses against *P. falciparum *antigens, varying from low to high B and T cell responses and indicating that the immune response to most of antigens can be used to assess malaria transmission in epidemiological surveys. Very few of such studies show an association with clinical immunity [65-74].

Evidence that the protective immunity to *P. falciparum *malaria is associated with different classes and subclasses of antibodies reveals the importance of considering the quality of the response. In Brazil, analysis of the antibody isotypes specific for several *P. falciparum *proteins revealed that all four IgG subclasses are present and, for some proteins such as the N-terminal region of the p126 protein, individuals with higher levels of anti-Nt47 cytophilic IgG antibody had significantly lower parasitaemia levels [68]. In contrast, plasma concentrations of IgG against a detergent-soluble extract of *P. falciparum *schizonts, the concentrations of anti-parasite antibodies of all subclasses increased with age. There was no correlation between age and the proportion of cytophilic antibodies [75] and no major difference was observed in IgG subclass distribution of antibodies to the polymorphic block 2 and the 19-kDa C-terminal domain MSP-1 between symptomatic, and asymptomatic parasite carriers [71].

The presence of asymptomatic infections in Amazonian communities suggests that, in fact, a similar phenomenon may be occurring in areas of low *P. vivax *transmission in Brazil. The gradual nature of the acquisition of this immunity is known to be partially mediated by malaria specific antibodies responses, since passive transfer of purified immunoglobulin from immune individuals rapidly reduces the recipient's parasitaemia [76]. Despite this, the factors that govern the development of acquired immunity after natural infection remain poorly understood. The identification of parasite antigens that induce protective antibody and cellular responses would be an important step toward understanding of naturally acquired immunity to *P. vivax *malaria.

In Brazil, naturally occurring antibody to *P. vivax *sporozoites have been reported since the late 1980s in indigenous population in the State of Para Brazil [77]. Since then, polymorphism of the *P. vivax *CS protein of *P. vivax *was reported and, in addition to parasites with the original CS repeat amino acid sequences, designated *P. vivax *VK210, a variant VK247 and *P. vivax*-like was also described [78,79]. The human *P. vivax*-like parasite has a CS repeat which corresponds to the simian parasite *P. simiovale*. Otherwise the identity of these parasites is not clear, since a line of the human isolates has not yet been obtained and the clinical manifestation of this infection, as well as that of VK247 variant is unknown.

In view of this finding, blood samples from the indigenous population mentioned above were re-examined and high level of reactivity against the variants were detected. These results demonstrated that these variants have been present in Amazonian Indians for at least 12 years [80]. Other studies showed the occurrence of *P. vivax *variants both by serology and PCR reaction in samples from different endemic regions in Brazil [14,48,81,82]. In non-endemic regions, *P. vivax *and variants have been also detected.

Studies on the naturally acquired humoral immune responses to several *P. vivax *blood stage vaccine candidates, such as PvMSP-1 (Merozoite Surface Protein 1), PvAMA-1 (Apical Membrane Antigen 1), PvMSP-3 (Merozoite Surface Protein 3); PvMSP-9 (Merozoite Surface Protein 9, RBP (Reticulocyte Binding Protein) and DBP (Duffy Binding Protein) show that they are immunogenic in distinct Brazilian endemic areas with different levels of exposure [63,74,83-91]. The specific antibodies induced by natural infections to most of the proteins are associated with time of exposure in endemic regions, a phenomenon which has been frequently reported for various antigens reflecting most likely exposure to the parasites and possibly maturation of the immune system over time. Although there is consistent evidence from human and animal model system that cell-mediated immunity may contribute both to protection and to pathogenesis, the knowledge on cellular immune response in vivax malaria and the factors that may regulate this immunity are still poorly understood [92]. Very few studies have been able to find a clear association between specific cellular and antibodies to vaccine candidates and protection. However, direct comparison of natural cellular and antibodies responses to *P. vivax *antigens may provide valuable insight as to how a malaria vaccine might work. Probably, an accumulation of a comprehensive repertoire of antibodies recognizing antigenically distinct molecules may be the key to acquisition of clinical protection.

### Malaria research and teaching in Brazil

A survey of the groups working on different aspects of malaria in Brazil was made in 2007 to facilitate and promote contacts with malariologists outside Brazil [93]. A total of 70 groups were identified. From these, 46 were considered to be effectively involved in conducting research on malariology. Groups working with other approaches, such as clinical, control, information, prevention and therapeutics, without being, however, directly concerned with research have also been listed. The catalogue included a comprehensive classification of the groups, according to the themes of interest and expertise (considering that a group could be competent and interested in more than on subject. Five groups were working on biochemistry (including organic synthesis and medicinal chemistry), five on cell biology, 15 on clinical aspects, nine on diagnosis, 30 on drugs and therapeutics (natural products, perspectives for drug development, pharmacology, prophylaxis, chemo-resistance and response monitoring), 17 on entomology and vector control, 34 on epidemiology and control, 16 on genetics and molecular biology (including recombinant proteins and genetic polymorphisms), 19 on immunology (immune protection, immune response, immunomodulation, immunopathology and vaccine development and testing in preclinical trials), five on parasitology and six on pathogenesis and pathology.

It is important to point out that only 19% of these research centres are located in the Amazon region (Figure 6). Despite occupying more than half (58%) of the country territorial surface (approximately 8,511,965 square kilometers) and concentrating 99,8% of the registered cases of malaria, the region hosts only around 8% of the urban and 12% of the total population of the country, 10% of the electorate in Brazil and accounts for about 5 percent of the nation's gross national product [94]. A volume of the catalogue can be obtained by request to the corresponding author (CTDR).

Some teaching activities focusing specifically on malariology are offered to students and graduates in Brazil. Courses on host-parasite relationship, parasitology or tropical medicine, that cover malaria together with other parasitic and infectious diseases in the programme, are not considered here. A two-week course (including practical classes on parasitological examination of blood films) is offered yearly (in November) by the team of the CPD-Mal to post-graduate (PG)(MSc and PhD) students from the courses of IOC at Fiocruz and from other institutions [95]. A three-week course, also directed to PG students (Course on Infectious and Parasitic Diseases at the Faculty of Medicine of the University of São Paulo - FM-USP, takes place at a biennial rhythm at the Instituto de Medicina Tropical de São Paulo and is also opened to students and graduates from other institutions [96]. A one-week course on control of malaria vector, with emphasis on biological control and practical field activities is offered yearly at the Instituto Nacional de Pesquisas da Amazônia (end of October beginning November)[97].

The *Seminário Laveran & Deane sobre Malária *is organized yearly by the CPD-Mal/Fiocruz, since 1995, to analyze and improve the project thesis of MSc and PhD Brazilian students. The participants are confined in a hotel (on the Itacuruçá Island) for five days, when the projects are presented and discussed both in plenary and in group discussion sections. Each student has two senior tutors that follow the student for the duration of the seminar, helping him/her in the composition of his/her final report. The body of Professors also proposes a final Senior Report to each student. The *Seminário *is supported mainly by the PNCM (as well as by the CNPq and Faperj, and has also financial help from the French Government and the Sanofi-Aventis Impact Malaria Initiative). The full methodology and a detailed description of the dynamics of the *Seminário *can be found at its website [95].

## Competing interests

The authors declare that they have no competing interests.

## Authors' contributions

JOF summarized the work done on malaria immunity and data on malaria in the Atlantic Forest and wrote the corresponding texts, prepared the final presentation of figures and organized the final text together with CTDR, MVGL worked with data on severe malaria and on hospitalized patients in the Amazon Region and wrote, together with JLBL and PT the perspectives on control of the disease, PB worked mainly with data concerning outpatient clinics and hospitalized patients from the Extra-Amazon region and data on autochthonous malaria in the Atlantic Forest, in Rio de Janeiro, JLBL and PT provided data on the present situation as well as historical information about malaria in the Country. CTDR planned and coordinated the article, choose the figures, dealt with data on immunity, clinical and historical aspects and reviewed and organized the final document with JOF. All authors read and approved the final manuscript.
